# Acute Functional Gastric Outlet Obstruction Associated With Low-Dose Tirzepatide

**DOI:** 10.7759/cureus.78090

**Published:** 2025-01-27

**Authors:** Mina Iskander, Manish Wadhwa, Yeongjin Kim, Neha Singh, Prutha Pathak

**Affiliations:** 1 Internal Medicine, North Alabama Medical Center, Florence, USA

**Keywords:** diabetes, gastric outlet obstruction (goo), obesity, tirzepatide, weight loss

## Abstract

Obesity has become a significant global challenge, highlighting the need for effective weight loss strategies. Glucagon-like peptide-1 receptor agonists (GLP-1 RAs) have emerged as a promising solution, with tirzepatide gaining significant popularity for its efficacy in promoting weight loss. This case report highlights a 57-year-old male patient with type 2 diabetes on a low dose of tirzepatide who initially presented with severe abdominal pain and vomiting, with symptoms resembling those of acute functional gastric outlet obstruction. Despite initial suspicion of an anatomical obstruction, no abnormalities were found on esophagogastroduodenoscopy. The symptoms resolved following the discontinuation of tirzepatide, suggesting a causative link. This case underscores the importance of physician awareness and patient education regarding potential severe gastrointestinal side effects associated with tirzepatide, even at low doses.

## Introduction

Glucagon-like peptide-1 receptor agonists (GLP-1 RAs) are gaining increasing popularity for their benefits in weight loss. Initially developed as a treatment for type 2 diabetes, their advantages have expanded to include associations with reduced cardiovascular and all-cause mortality [1]. Furthermore, these medications hold the potential to treat metabolic dysfunction-associated steatotic liver disease (MASLD) and inflammatory bowel disease (IBD) [2]. Among GLP-1 RAs, tirzepatide (a dual glucose-dependent insulinotropic polypeptide (GIP) and GLP-1 RA) has garnered significant attention following its FDA approval for weight loss in November 2023. The most common adverse events associated with GLP-1 RAs are gastrointestinal symptoms, which include nausea, vomiting, diarrhea, and constipation. These symptoms are typically mild to moderate, transient, and often occur during dose escalation [3]. We present a unique case of acute functional gastric outlet obstruction linked to a low dose of tirzepatide.

This case was previously presented as a poster at the American College of Gastroenterology on October 27, 2024. An online abstract only was published in a supplement version of the *American Journal of Gastroenterology* in October 2024. No full manuscript/case report has been submitted or published anywhere else. This is the only submission for the full manuscript.

## Case presentation

A 57-year-old male patient with a history of type 2 diabetes and hypertension presented to the emergency department with severe abdominal pain and vomiting. The pain began approximately four weeks prior to presentation and has progressively worsened since. The abdominal pain was described as colicky, primarily located in the right lower quadrant, and radiated to the right flank, with a severity of eight out of 10. The patient also reported multiple episodes of non-bloody vomiting that occurred more frequently after eating, which subsequently led to a decreased appetite. The symptoms were accompanied by altered bowel habits, including intermittent episodes of diarrhea. He had experienced abdominal discomfort and nausea several months prior, but these symptoms were mild and tolerable at that time. He presented to the emergency department two months ago with complaints of mild abdominal pain and was discharged to follow up at an outpatient clinic after his laboratory tests and imaging results were within normal limits. His home medications include tirzepatide 2.5 mg subcutaneous injection once weekly, glipizide 5 mg daily, losartan 100 mg daily, and tadalafil once daily. On physical examination, he appeared dehydrated with sunken eyes and signs of pain, presenting with a markedly distended abdomen that was diffusely tender, especially on the right side, while bowel sounds were present. Initial vitals showed blood pressure of 160/90 mmHg and heart rate of 92 bpm, with 97% saturation on room air. Laboratory tests showed a white blood cell count of 11.8 x 10³ cells/µL with 68% neutrophils, hemoglobin of 14.1 g/dL, hematocrit of 41.1%, and platelet count of 240 x 10³ cells/µL. A complete metabolic panel revealed sodium at 139 mEq/L, potassium at 3.9 mEq/L, chloride at 102 mEq/L, bicarbonate at 26 mEq/L, and an anion gap of 11 mEq/L. The renal function tests indicated blood urea nitrogen (BUN) of 15 mg/dL and a creatinine level of 1.0 mg/dL. Additionally, the glucose level was 166 mg/dL, with hemoglobin A1c (HbA1c) of 6.2%. Liver function tests showed aspartate aminotransferase (AST) at 31 U/L and alanine aminotransferase (ALT) at 30 U/L, along with a total bilirubin level of 0.5 mg/dL and a lipase level of 92 U/L.

**Figure 1:**
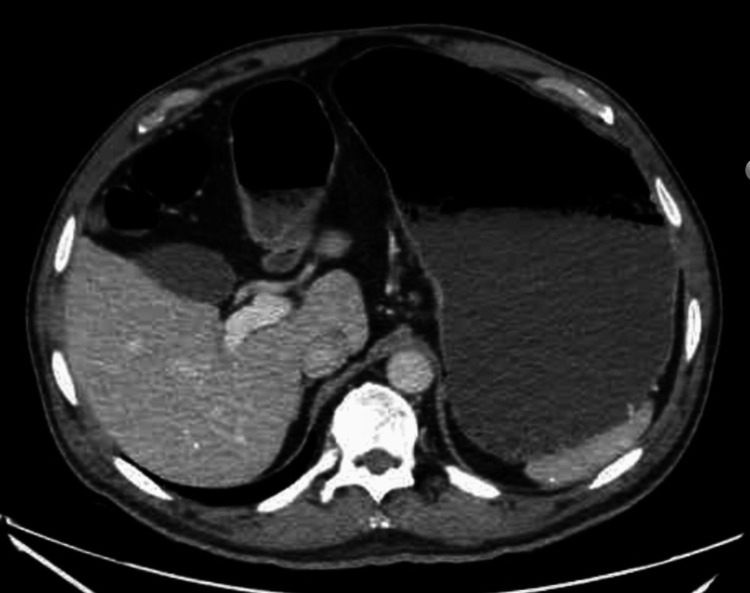
CT of the abdomen with contrast showing significant gastric distention and fluid retention

**Figure 2:**
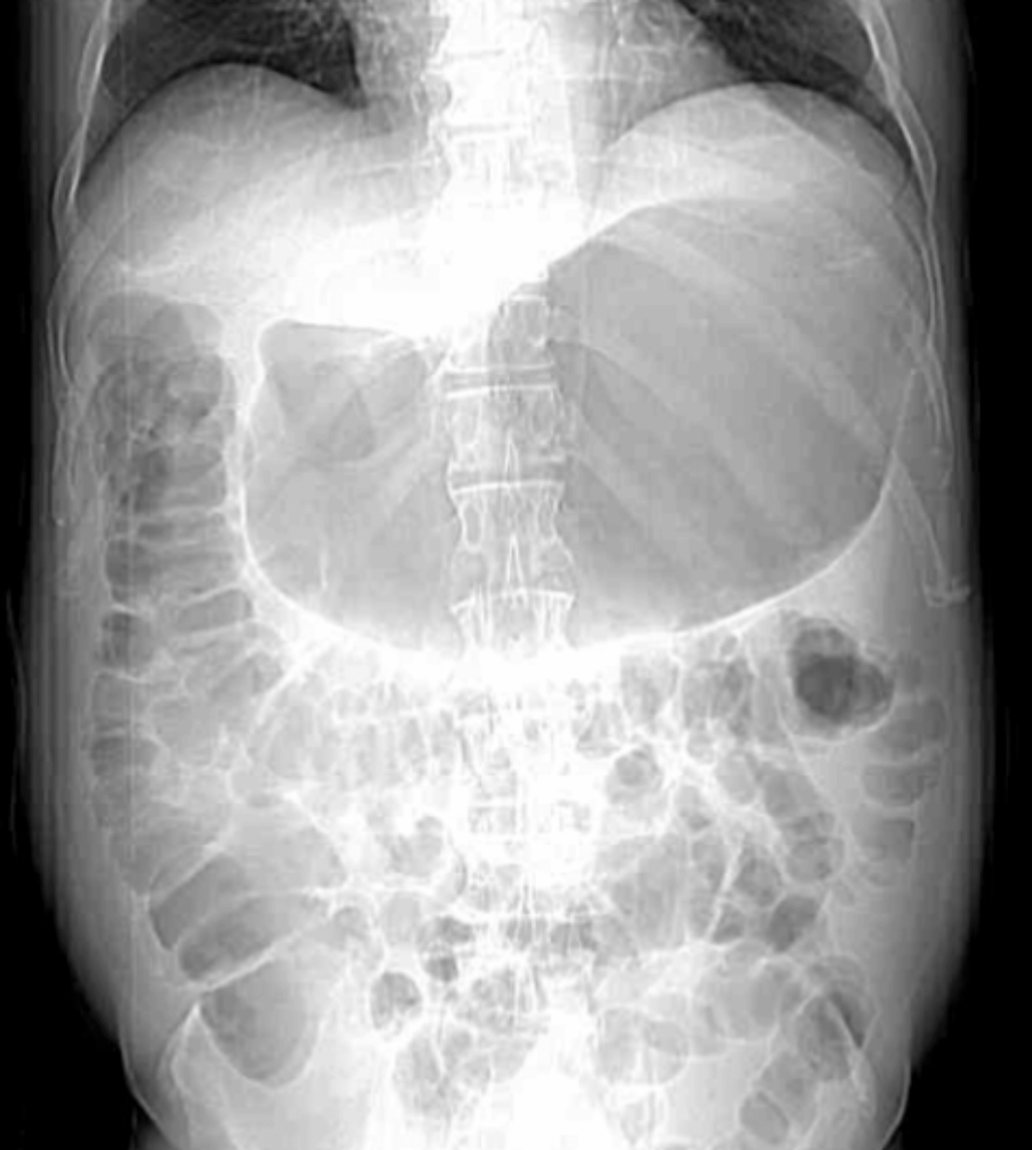
CT of the abdomen and pelvis showing markedly distended stomach

An abdominal ultrasound was performed, which showed no significant abnormalities; however, a computed tomography scan of the abdomen and pelvis with intravenous contrast revealed a markedly distended stomach with air-fluid levels and food debris, suggesting gastric outlet obstruction (Figures 1, 2). The patient was managed supportively with the insertion of a nasogastric tube connected to low intermittent suction for bowel decompression along with intravenous fluids, mainly lactated ringer. He was evaluated by the gastroenterology team the following day. Given the severity of his presentation, there was a suspicion of an anatomical obstruction, such as an ulcer or malignancy. Esophagogastroduodenoscopy (EGD) was performed on the third day of admission, and no pathological findings or obstructions were identified. An oral diet was gradually introduced as tolerated. Upon further discussion, the patient explained that his symptoms had started shortly after being switched from metformin to tirzepatide, and he was reluctant to stop the drug because it had helped him lose weight. Tirzepatide was discontinued at the time of discharge, and he reported no recurrence of symptoms during follow-up.

## Discussion

Tirzepatide is the only FDA-approved drug in the class of dual GIP/GLP-1 RAs, which, although not exclusively GLP-1 agonists, have similar pharmacological properties [1]. GLP-1 RAs are known to cause gastrointestinal side effects, most commonly nausea, vomiting, diarrhea, and constipation. They are usually transient, typically starting during the dose escalation period and generally resolving shortly after the maintenance dose is reached [3]. In patients with type 2 diabetes, GLP-1 RAs have been linked to an increased risk of more severe complications, including intestinal obstruction, with the risk reaching up to 3.5-fold in one study [4]. Another associated complication is gastroparesis. However, severe gastroparesis leading to acute gastric dilatation and functional gastric outlet obstruction has been rarely reported. The cases we identified were associated with liraglutide and semaglutide [5,6].To our knowledge, this may be the first reported case of acute functional gastric outlet obstruction associated with tirzepatide. In our patient, we attributed the symptoms to tirzepatide rather than diabetes-induced gastroparesis, as his diabetes was well controlled, evidenced by an HbA1c of 6.2%. Furthermore, the onset of symptoms shortly after starting the drug and their resolution following discontinuation support this association. The mechanisms include action on gastric neuronal cells and delayed gastric emptying, which may lead to an increase in retained gastric contents [7]. This raises concerns about an increased risk of aspiration, whether perioperatively or during endoscopy. Several studies have demonstrated a higher risk of retained gastric contents with the use of GLP-1 RAs [8]. Nadeem et al. conducted a retrospective study with 35,183 patients who underwent EGD and found that the use of GLP-1 RAs was associated with a fourfold increase in gastric content retention, a fourfold rise in aborted EGDs, and a doubling in the need for repeat procedures, even when adjusting for diabetes [9]. A systematic review and meta-analysis of studies that focused on quantified metrics of gastric delay revealed an ∼36-minute gastric emptying delay with GLP-1 RA medications, which is minimal compared to standard fasting periods, and showed no significant differences in liquid emptying at time points relevant to periprocedural care [10]. However, despite an increased risk of retained gastric contents, there is not enough evidence to confirm an increased risk of aspiration, but there have been reported cases of aspiration [11]. In our case, EGD was done on the third day of admission, and the patient has been on the nasogastric tube with suction for two days. No retained gastric contents were found. In an effort to avoid risk of aspiration, a multi-society clinical practice guideline for perioperative use of GLP 1 RA was released by the American Gastroenterological Association (AGA), the American Society for Metabolic and Bariatric Surgery (ASMBS), the American Society of Anesthesiologists (ASA), the International Society of Perioperative Care of Patients with Obesity (ISPCOP), and the Society of American Gastrointestinal and Endoscopic Surgeons (SAGES). Recommendations emphasize shared decision-making among patients and healthcare teams, individual risk assessment for delayed gastric emptying and aspiration, and tailored management strategies. Patients at low risk can continue GLP-1 RA therapy, while those with concerning risk factors should adopt a preoperative liquid diet for at least 24 hours. Point-of-care gastric ultrasound may be another useful tool to evaluate aspiration risk [12]. Another concerning factor is that some patients may be reluctant to stop the medication despite severe side effects. That is because many individuals seeking weight loss often do so with a primary focus on esthetic or personal satisfaction rather than health outcomes [13]. Therefore, physicians should take the time to thoroughly educate patients about the use of GLP-1 RAs, including tirzepatide, along with their common side effects, and review any signs and symptoms during follow-up.

## Conclusions

Tirzepatide provides a lot of health benefits with a favorable safety profile. Gastrointestinal side effects are common and are usually well tolerated. However, in severe cases, they can lead to serious side effects including those secondary to delayed gastric emptying. As tirzepatide is increasingly prescribed and our understanding of it expands, physicians should recognize that its side effects may not be strictly dose-dependent and can manifest even at lower doses. Additionally, they should stay informed about the latest practice guides to mitigate the risk of severe complications. Additional research is required to ascertain the precise risks associated with its use. Moreover, physicians should thoroughly educate patients about the side effects of tirzepatide, especially those that have other risk factors of delayed gastric emptying.
